# The influence of molecular shape on reorientation dynamics of sizable glass-forming isomers at ambient and elevated pressure

**DOI:** 10.1038/s41598-023-50894-8

**Published:** 2024-01-09

**Authors:** Alfred Błażytko, Marzena Rams-Baron, Marian Paluch

**Affiliations:** August Chełkowski Institute of Physics, University of Silesia in Katowice, 75 Pułku Piechoty 1, 41-500 Chorzow, Poland

**Keywords:** Physics, Atomic and molecular physics, Condensed-matter physics

## Abstract

We used dielectric spectroscopy to access the molecular dynamics of three isomers with a structure based on a sizable, partially rigid, and non-polar core connected to a polar phenylene unit differing in the position of the polar group, and, consequently, the direction and magnitude of the dipole moment to address the question how unique molecular properties, in particular large size and elongated shape, affect the dynamics. The position of the polar group differentiates the molecular shape and isomer’s anisotropy and leads to different thermal and dynamic properties of the isomers. The shape of permittivity loss spectra was governed by magnitudes of the longitudinal and transverse components of dipole moment to a large extent. For para isomer with negligible traverse component of dipole moment, the narrowest loss peak was found while for meta isomer, the bimodal loss peak was observed at high temperatures. Its shape evolved on cooling limiting the possibility of individual mode separation near glass transition where the dynamics were more cooperative. High-pressure dielectric studies showed that sizable isomers were characterized by the pronounced sensitivity of glass transition temperature, T_g_, to compression. Observed high activation volumes, such as 735 cm^3^/mol at T_g_ for para isomer, were found to correlate with the length scale of dynamic cooperativity. The number of dynamically correlated molecules depended on molecular shape and varied among isomers while the determined values were much smaller than that reported for other glass-forming liquids. We discussed here the obtained results in the context of the specific properties of the systems studied showing the overriding role of anisotropy.

## Introduction

The study of molecular mobility is important in many areas of science and engineering, including materials science, physics, and chemical engineering. It can provide insight into the physical and chemical properties of materials and can help to design and optimize materials for specific applications. Broadband dielectric spectroscopy (BDS) is one of the most common techniques for molecular dynamics examination with a longstanding tradition of studying supercooled liquids and glasses. What constantly attracts researchers' attention to glass-forming materials is the spectacular evolution of dynamic properties covering 15 orders of magnitude in a relatively small temperature range between the melting and glass transition temperatures, providing unique opportunities to study the fascinating and still not fully understood effects related to the cooperative phenomenon of liquid-to-glass transition.

To study the reorientation dynamics using the BDS method, the molecule must exhibit a non-zero value of the dipole moment^1^. The case of anisotropic glass-forming molecules is of particular interest because the longitudinal and transverse components of the dipole moment can contribute to the dielectric response to a different extent. In contrast, for small and symmetrical particles with isotropic shapes, such differences are expected to be negligible. Most anisotropic glass-forming molecules investigated to date host rod-shaped, disc-shaped, or bent-core structures that can form liquid crystalline phases. The importance of molecular shape in determining their mesogenic and dielectric properties has been well recognized^2^. In the vast majority of these materials the dipole moment was directed along the long molecular axis, making the longitudinal component of a dipole moment a main probe of molecular motion. On the other hand, very few studies have concentrated on the role of anisotropy in the dielectric response of disordered systems. Consequently, our understanding of how the longitudinal and transverse components of dipole moment sample different aspects of reorientations in such anisotropic glass-formers is still incomplete.

Recently, unusual dielectric behavior at a high temperature was reported for anisotropic glass formers with a sizable core^3^. When most supercooled liquids at high temperatures revealed a narrow and symmetric Debye-like dielectric loss peak which broadened on cooling, a representative of sizable molecules showed the bifurcation of the main loss peak at high temperatures. This behavior has been interpreted as a manifestation of individual aspects of the motion of a sizable and anisotropic molecule, which can reorient along short and long axes on different time scales. Of course, this result raises the question of how exactly the dielectric response i.e. bimodal peak shape is connected to molecular properties of sizable glass-formers.

It is generally agreed that as the glass transition is approaching, the character of molecular motions becomes more cooperative^4^. The independent reorientations observed at high temperatures, evolve on cooling towards cooperative behavior where the motion of molecules depends strongly on their neighborhood. Therefore, as the temperature increases towards the melting temperature the shape of dielectric spectra, sensitive to dynamic heterogeneity, can uncover some individual features of molecular motion whose discrimination near T_g_ is unattainable. On the other hand, molecular dynamics, structure, and properties (also cooperativity) of glass-forming materials can be also effectively tuned by varying the pressure at a constant temperature. By performing dielectric studies at elevated pressure, it is possible to determine the activation volume characterizing the sensitivity of reorientation dynamics to compression^5^. Its value depends on the size of the reorienting unit, but in the case of isomers with identical molecular weights, any differences may shed more light on the importance of anisotropy and molecular shape. In the case of molecules with elongated shapes, the application of pressure can provide additional information to understand better the eventual separation of time scales of relaxations associated with long and short axes reorientation and differences in pressure sensitivity of particular modes. Additionally, the relation between activation volume and cooperativity length scale for structural relaxation was reported^6^ which validity is interesting to check in the context of unusual properties found for sizable glass-formers in the previous studies^3,7^.

**Figure 1:**
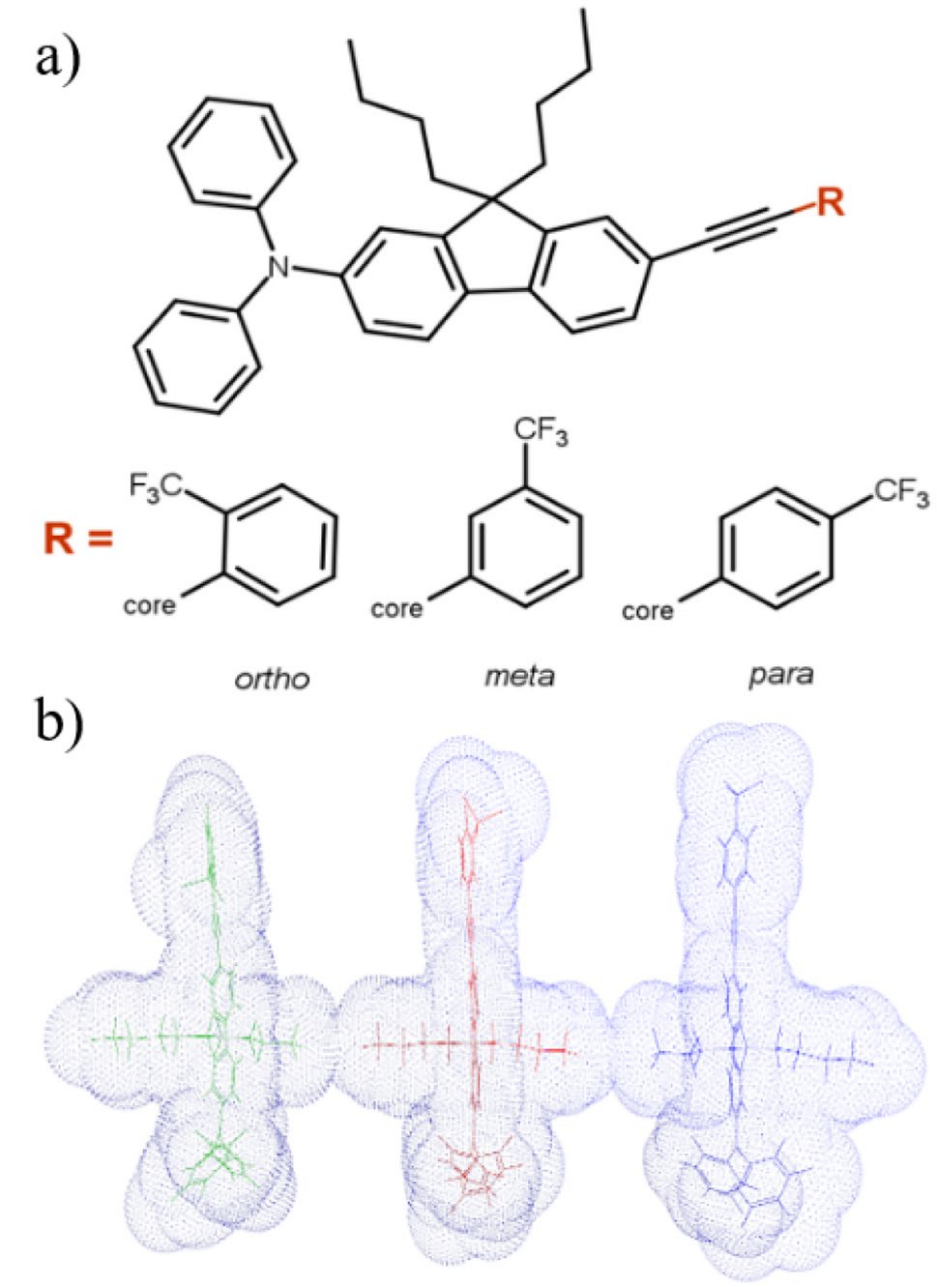
(**a**) The chemical structure of the studied isomers. (**b**) Geometrically optimized structures through the AM1 semi-empirical method. Starting from the left: ortho-CF_3_, meta-CF_3_, and para-CF_3_ are presented. Symbolic mesh shows the van der Waals volume of the molecules. The drawing was created using Vega ZZ software.

A first indication of the bimodal dielectric response of sizable glass formers was given in ref.^3^. In our considerations, the term sizable glass formers refers to molecules with a molecular weight above 600 g/mol that have multiple rigid ring-containing structures and exhibit unique dielectric properties e.g. large value of pre-exponential factor of a Vogel–Fulcher–Tammann (VFT) law used to describe the temperature dependence of an α relaxation process originated from reorientation around shorter molecular axis^3^. Here, we investigated other representatives of sizable glass-formers with a structure based on the same concept of an anisotropic, partially rigid, and sizable non-polar core containing various heterocyclic structures and aliphatic chains connected to a polar phenylene unit, see Fig. 1. As a linker between polar and non-polar segments, we used an acetyl bridge, unlike in the previous paper^3^. The linker modification allows us to increase the system anisotropy in comparison to sizable glass-formers investigated before. We used a trifluoromethyl group, –CF_3_, to introduce the dipole moment to the investigated molecules. It was variously attached to the phenyl ring at the ortho, meta, or para positions. This approach provided us with isomers with a well-defined direction of the dipole moment vector and different molecular shapes – more elongated in comparison to those studied in ref.^3^, suitable for further systematic dielectric studies of their reorientation dynamics.

The main goal of the current work was to address the question of how differences in molecular shapes among isomers will impact the spectral properties revealed in dielectric investigations at ambient and elevated pressure in thermodynamic conditions corresponding to a supercooled liquid state. To facilitate understanding of the complex reorientation dynamics above the T_g_, we analyzed and discussed data collected below T_g_, where the relaxation pattern also depends on the position of the –CF_3_ group attached to the rotating phenyl ring. Our calorimetric and dielectric studies revealed many differences in thermal and relaxation properties between isomers which were largely due to differences in the molecular shapes. At the same time, certain characteristic features, common to all isomers were identified. Namely, high-pressure studies showed that all isomers revealed pronounced sensitivity of reorientation dynamics to compression as indicated by very high values of activation volumes and pressure coefficients of T_g_. A particularly important result of this work was the observation of complex dielectric response for a meta isomer at high temperatures and at elevated pressure which we attributed to reorientation along the long and short molecular axes whose time scales decouple as the degree of intermolecular cooperativity decreases. In addition, we examined the degree of heterogeneity near the glass transition and confirmed the validity of a relation between the correlation radius and the activation volume reported for other glass-forming materials^8^. We discussed the obtained results in the context of the specific properties of isomers studied—their large size, substantial stiffness of sizable cores, and elongated shape. We have shown the overriding role of anisotropy which we identified as a pivotal feature determining the dynamic properties of the studied isomers.

## Results and discussion

### Thermal characterization

**Figure 2:**
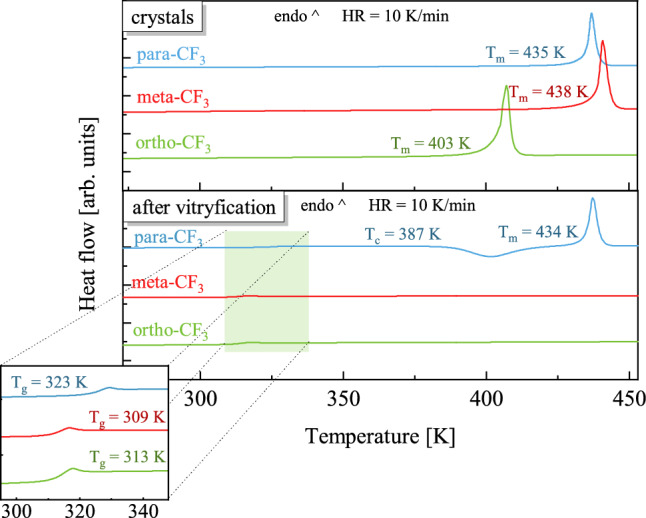
Thermograms showing thermal processes observed during heating of crystalline (upper panel) and vitrified (lower panel) samples. The inset magnifies the glass transition region.

Figure 2 shows thermograms measured on heating of crystalline (top panel) and vitrified (bottom panel) isomers of sizable molecules. On thermograms recorded for crystalline samples only one endothermic process was observed (top panel), which corresponds to the melting of the crystalline material. The highest melting temperature, T_m_, was found for meta-CF_3_, i.e. T_m_ = 438 K. The para and ortho isomers melt at T_m_ = 435 K and T_m_ = 403 K, respectively. The T_m_ value observed for ortho-CF_3_ was found to be significantly lower in comparison to other isomers, which may indicate the presence of weaker bonds in the crystal structure of the ortho isomer^5^.

A step change in heat capacity, indicating the glass transition phenomenon, was observed on each thermogram recorded on heating of vitrified samples. The exact temperature values at the midpoint of the glass transition were determined using temperature-modulated differential scanning calorimetry (TMDSC). The highest value of the glass transition temperature, T_g_, was found for para-CF_3_ i.e., T_g_^TMDSC^ = 323 K. The ortho and meta isomers show the glass transition at T_g_^TMDSC^ = 313 K and T_g_^TMDSC^ = 309 K, respectively. Several factors can affect the glass transition temperature, such as differences in molecular structure (molecular weight, polarity, shape) and the ability to form intermolecular bonds^9–12^. The studied isomers have the same mass and atomic composition. However, due to the differences in the position of the CF_3_ group, the shape of a molecule and the strength of intermolecular interactions can vary among isomers.

The thermogram registered for para-CF_3_, besides the glass transition feature, shows an additional process at T_c_ = 387 K attributed to non-isothermal crystallization. The lowest physical stability of vitrified para-CF_3_ among investigated isomers is related to its higher symmetry facilitating the formation and growth of crystals. As shown on several low-molecular-weight liquid pairs (like p-xylene and m-xylene, or benzene and toluene), those showing greater symmetry had a greater tendency to recrystallize^13,14^.

### Reorientation dynamics at ambient pressure

**Figure 3:**
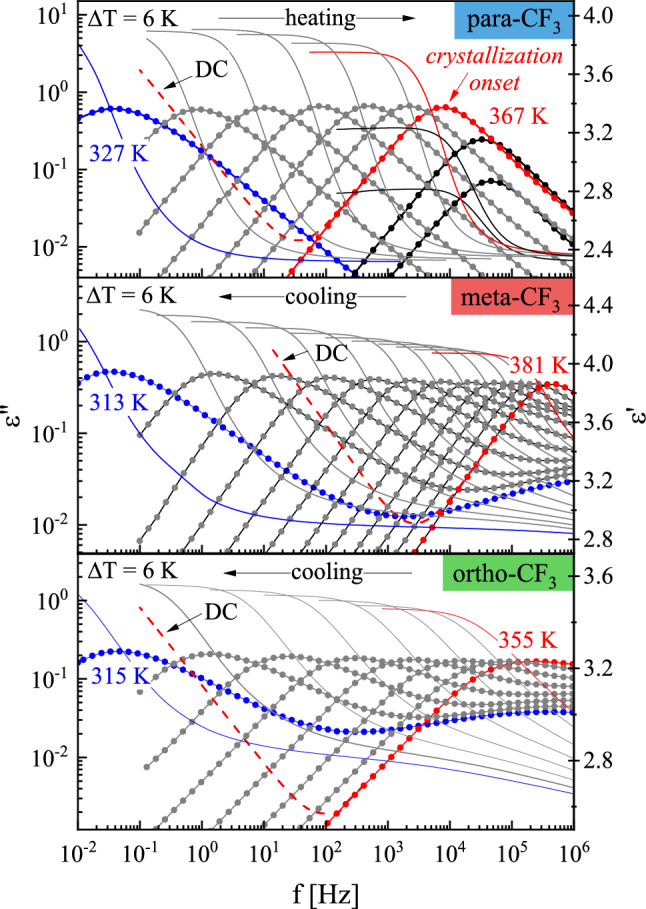
The dielectric response of para-CF_3_, meta-CF_3,_ and ortho-CF_3_ at ambient pressure. The solid lines represent the real part of dielectric permittivity ε’ (right axis), while the dotted lines indicate the imaginary part of the permittivity ε” (left axis). In the case of para-CF_3_ black color was used to distinguish partially recrystallized spectra. The dc-conductivity has been removed from the spectra, except one illustrating the magnitude of dc contribution (dashed red line).

To characterize the reorientation dynamics and compare the properties of particular isomers at ambient pressure we performed first the dielectric measurements at temperatures corresponding to the supercooled liquid state. Figure 3 shows the frequency dependence of the imaginary part of complex permittivity, ε"(f), at various temperatures. The prominent process well-resolved on all recorded ε"(f) spectra is a structural relaxation peak (denoted also as α-relaxation) corresponding to the cooperative reorientation of the molecules and directly related to the glass transition. For anisotropic elongated systems, such neighbor-dependent molecular rearrangement can occur via long or short axes reorientations. On cooling the structural relaxation peak maximum shifts toward lower frequencies manifesting a growing time-scale required for molecular reorientation as the temperature decreases towards the glass transition. In contrast to meta-CF_3_ and ortho-CF_3_, the amplitude of α-relaxation for para-CF_3_ started decreasing at T = 367 K. Such behavior denotes the onset of crystallization and is consistent with the behavior revealed in DSC studies indicating recrystallization of the para-CF_3_ sample when heated above T_g_. When liquid–crystal transformation progresses on heating, para-CF_3_ molecules that constitute the crystal lattice lose the ability to reorient. As a consequence, the decreasing number of mobile dipoles is accompanied by a drop in the α-peak amplitude^15^.

**Figure 4:**
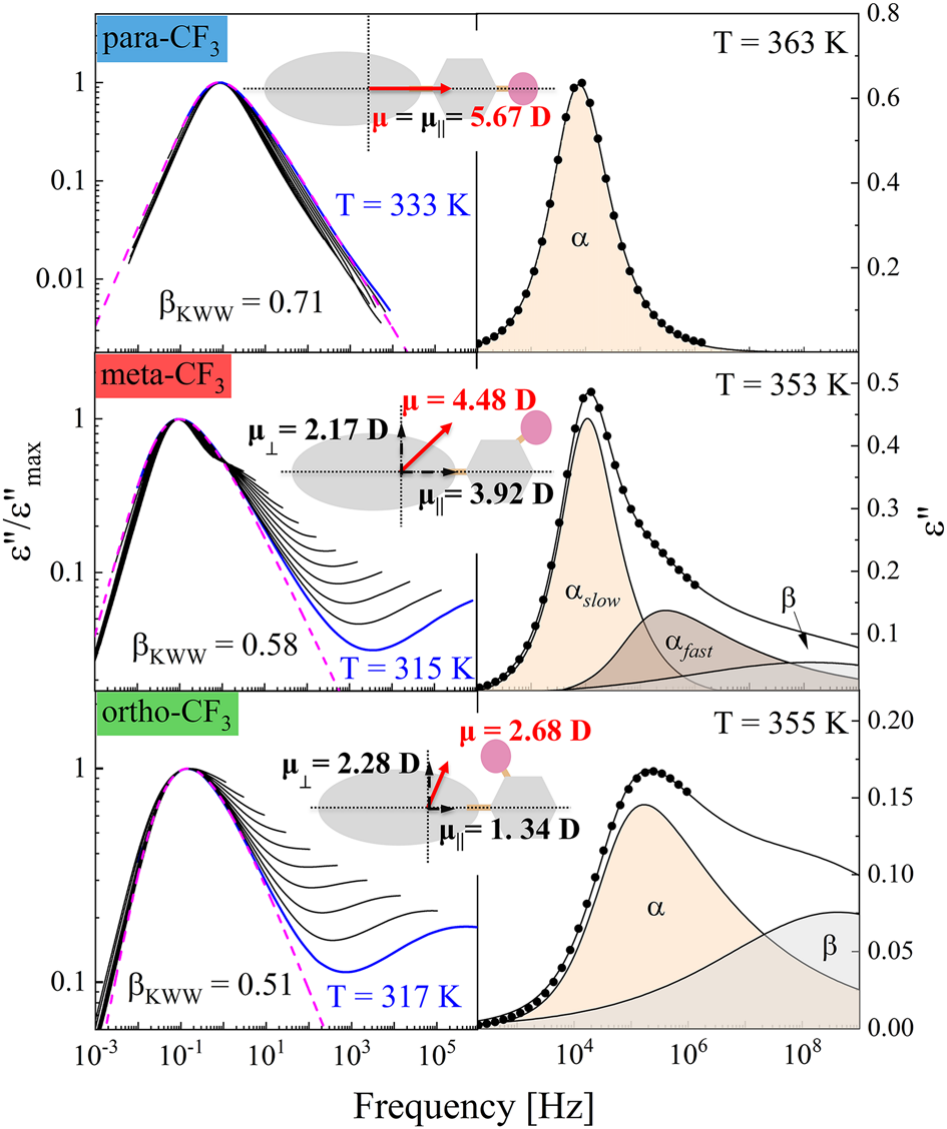
Left panel: Masterplots (superimposition of spectra on selected ones) with the applied KWW function (dashed line). The insets of each plot show a schematic representation of the molecule and dipole moment vectors with dipole moment values in parallel and perpendicular axes. Right panel: Example spectrum with HN fit function for para-CF_3_ and meta-CF_3_, and with HN and CD fit functions for ortho-CF_3_.

To get more detailed information about the relaxation properties of the isomers studied, the data presented in Fig. 3 were fitted using the Havrilik–Negami (HN) function with the dc-conductivity term:

$$\varepsilon^{*} = \frac{\sigma }{{\varepsilon_{0} \omega }} + \varepsilon_{\infty } + \frac{\Delta \varepsilon }{{\left( {1 + \left( {i\omega \tau_{HN} } \right)^{\alpha } } \right)^{\beta } }}$$

where $\sigma /\varepsilon_{0} \omega$ defines the dc-conductivity contribution, ε_∞_ is the high-frequency permittivity limit, Δε is dielectric strength, ω is the angular frequency, τ_HN_ corresponds to the Havriliak-Negami relaxation time, and the exponents α and β are the shape parameters^16^. The outcome of the fitting procedure for representative spectra selected for each isomer is shown in the right panels of Fig. 4.

For meta and ortho isomers, apart from the main structural relaxation peak, the additional contribution from the secondary relaxation denoted as the β-process was distinguishable in the high-frequency part of the spectrum. Although the maximum of β-process was far outside the experimental window its presence was included in the right panel of Fig. 4 to make the comparison among isomers clearer. The main experimental peak was satisfactorily fitted with a single HN function for para-CF_3_ and ortho-CF_3_ isomers, while for the meta-CF_3_, such an approach was insufficient. In the case of meta isomer, a strong shape asymmetry evolved on heating, indicating bifurcation of the primary process at high temperatures, required two HN functions. These two processes began to merge on cooling, and a single HN function could reasonably parametrize the ε"(f) data for meta-CF_3_ at temperatures below T = 340 K. A similar pattern of behavior was observed previously and attributed to various aspects of the motion of molecules with identical anisotropic sizable cores^3^. Due to their elongated shape, two molecular axes can be distinguished, relative to which the molecular reorientation can occur. Depending on whether the motion concerns the short or long axis, inertia impacts the reorientation to varying degrees. The link between the moment of inertia about the principal axes of the molecule, I, and the relaxation time about the same axes is hidden in the square-root relationship of the pre-exponential factor^17^ τ_0_ ∼ (2πI/k_B_T)^0.5^ in a function describing the nonlinear temperature behavior of relaxation times. Thus in the case of elongated molecules, reorientation with respect to the short axis is expected to be slower than reorientation about the longer axis being observed at higher frequencies^3^. The detection of mentioned aspects of reorientation depends on the presence of appropriate components of the dipole moment vector. A schematic illustration of each isomer with an indication of the components of the dipole moment vector is shown in the inset of Fig. 4. The presented values of the dipole moment, μ, were calculated using the AM1 semi-empirical method. For para-CF_3_ the calculated value was the highest, i.e. μ = 5.67 D, for the meta-CF_3_ μ = 4.48, and for the ortho-CF_3_ μ = 2.68 D. Variations in the magnitude of μ were reflected in the magnitude of dielectric strength Δε ~ Nμ^2^, where N is the number of mobile dipoles, as shown in Fig. 3, which was the lowest for ortho isomer. The dipole moment vector in the case of the para-CF_3_ was directed parallel to the long axis (the perpendicular component was negligibly small), so the movement of a molecule relative to the short axis dominated the dielectric response. In the case of meta-CF_3_ and ortho-CF_3,_ the dipole moment components parallel and perpendicular to the long molecular axis probe all reorientation aspects. So, both modes contribute to the dielectric response. If, due to the different impact of inertia effects, their time scales were different, we should be able to distinguish them in the dielectric response. The effect should be more pronounced at high temperatures where the mobility is less cooperative. This is particularly evident in the dielectric spectrum of the meta-CF_3_, where two relaxation processes were observed at high temperatures. We assigned them as α_*fast*_ (long axes reorientation probed by perpendicular component of μ) and α_*slow*_ (short axes reorientation probed by a parallel component of μ). In the case of the ortho isomer, such a clear indication was not found. This can be related to the much smaller magnitude of the parallel component of the dipole moment vector in ortho-CF_3_ (see illustration in inset to Fig. 4). Then the long-axis reorientations dominate the dielectric response and may cover the slower contribution to the α process. Nevertheless, both contributions will affect the width of the main relaxation peak in ortho-CF_3_ leading to its broadening.

To quantify the differences in the spectral shapes in terms of the frequency distribution of relaxation times between isomers, we created so-called masterplots by horizontally superimposing spectra recorded at different temperatures. The obtained masterplots depicted in Fig. 4 (left panel) showed significant differences in the shape of the loss peak between isomers. To quantitatively describe these differences, we applied a one-sided Fourier transform of the Kohlraush-Williams-Watt (KWW) function^18,19^:

$$\Phi \left( t \right) = exp\left[ { - \left( {\frac{t}{{\tau_{\alpha } }}} \right)^{{\beta_{KWW} }} } \right]$$

where β_KWW_ is a stretching parameter that takes values from 0 to 1, where a value of 1 indicates a narrow and symmetric α-relaxation peak (as in the Debye process); and the more β_KWW_ value approaches 0, the broader and more asymmetric the process is. The β_KWW_ value is commonly used to quantify dynamic heterogeneity, reflected in the frequency dispersion of relaxation times. The case of meta and ortho isomers investigated herein is unconventional because the shape of the dielectric loss peak results from two different reorientation modes, which, however, are indistinguishable near T_g_. Thus, the significance of the β_KWW_ parameter is not entirely the same as in the case of isotropic systems in which the dipole moment reflects the reorientation of the entire molecule. A possible approach for molecules investigated herein could be treating β_KWW_ value as a measure of the distribution of relaxation times resulting from individual contributions of distinct modes. Each isomer has a different α-relaxation shape regarding the frequency distribution of relaxation times reflected in distinct β_KWW_ values established for dielectric spectra collected close to T_g_. For para-CF_3_ β_KWW_ = 0.71 at T = 333 K, for meta-CF_3_ β_KWW_ = 0.58 at T = 315 K, and for ortho-CF_3_ β_KWW_ = 0.51 at T = 317 K. The highest β_KWW_ value in the case of the para isomer indicates the smallest distribution of relaxation times. The smallest degree of dynamic heterogeneity can be explained by the fact that in para-CF_3_ the dipole moment samples one type of motion, i.e., short axes reorientation. In contrast, for the ortho and meta isomers, the apparent broadening is due to two relaxation modes contributing to the main loss peak.

The next step was to find out the temperature dependence of relaxation times attributed to molecular reorientation. In particular, it was interesting to see differences in the time scale of reorientation along particular axes for the meta-CF_3_. Relaxation times τ_α_, τ_*slow*_ and τ_*fast*_ were determined from HN fit using the following formula^1^:

$$\tau = \tau_{HN} \left[ {sin\left( {\frac{\pi \alpha }{{2 + 2\beta }}} \right)} \right]^{ - 1/a} \left[ {sin\left( {\frac{\pi \alpha \beta }{{2 + 2\beta }}} \right)} \right]^{1/a}$$

**Figure 5:**
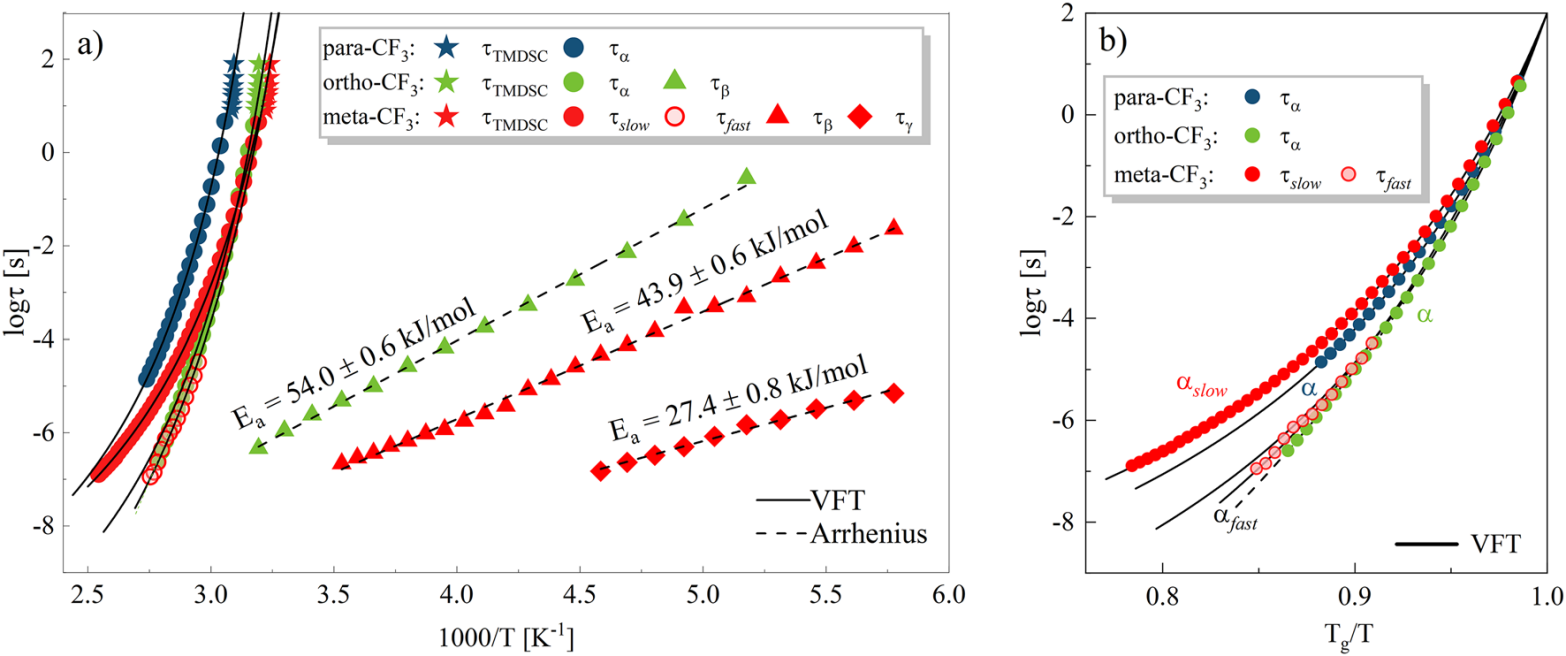
(**a**) Temperature dependence of relaxation times τ_α_ or τ_*slow*_ (closed circles) and τ_*fast*_ (open circles for meta-CF_3_) of three structural isomers: para-CF_3_ (blue), meta-CF_3_ (red), and ortho-CF_3_ (green). The asterisk denotes relaxation times determined from TMDSC. The solid line corresponds to the VFT fit function with fitting parameters denoted in Table 1. (**b**) Angel plot.

Figure 5 shows how the characteristic relaxation times evolve in a non-linear manner with temperature in the supercooled liquid phase. Such behavior can be parametrized by the Vogel–Fulcher–Tammann (VFT) function:

$$\tau_{\alpha } = \tau_{0} exp\left( {\frac{{DT_{0} }}{{T - T_{0} }}} \right)$$

where τ_0_ ∼ (2πI/k_B_T)^0.5^ is the pre-exponential factor depending on I according to Bauer expression^17^, D determines the deviation from Arrhenius behavior, and T_0_ is the so-called ideal glass temperature^20–22^. The value of fitting parameters τ_0_, D, and T_0_ are shown in Table 1. From the extrapolation of the VFT function to logτ_α_ = 2, the glass transition temperature, T_g_^BDS^, was determined as a temperature corresponding to a relaxation time of 100 s.

**Table 1:** Calorimetric and dielectric parameters characterizing dynamics of investigated isomers at ambient pressure conditions.

	T_m_ (K)	T_c_ (K)	T_g_^TMDSC^ (K)	T_g_^BDS^ (K)	β_KWW_	*m*	VFT parameters		
							logτ_0_ (s)	D	T_0_ (K)
para-CF_3_	435	387	323	322 ± 2	0.70	99 ± 12	− 12.31 ± 0.12	5.58 ± 0.14	275.56 ± 0.73
meta-CF_3_	438	–	309	308 ± 1	0.58	92 ± 5	− 11.77 ± 0.03	5.59 ± 0.05	262.02 ± 0.27
						102 ± 23^a^	− 16.05 ± 0.29^a^	9.12 ± 0.45^a^	252.78 ± 1.79^a^
ortho-CF_3_	403	–	313	310 ± 1	0.51	100 ± 8	− 16.53 ± 0.11	9.71 ± 0.17	252.85 ± 0.57

^a^Value for the α_*fast*_ process.

For the para-CF_3_ isomer, the glass transition temperature was T_g_^BDS^ = 322 ± 2 K, for meta-CF_3_ T_g_^BDS^ = 308 ± 1 K, and ortho-CF_3_ T_g_^BDS^ = 310 ± 1 K. The T_g_ values obtained from BDS and TMDSC were in very good agreement. In the case of the meta-CF_3_, the growing cooperativity of molecular dynamics on cooling is manifested in the course of VFT curves for α_*slow*_ and α_*fast*_ processes that merge when the T_g_ is reached. The closer to T_g_, the more difficult is to distinguish the different aspects of meta-CF_3_ motion. A similar effect related to the vanishing bimodal character of the loss spectra due to the cooperativity onset was observed recently by Blochowicz et al. for lower molecular-weight liquids^23^. To describe the temperature dependence of relaxation times for the ortho-CF_3_ with physically reliable parameters, we used two VFT functions. The limits of their applicability were determined from the Stickel analysis^24^. For meta-CF_3_ and para-CF_3_ the values of pre-exponential factor obtained from the free VFT fit of τ_α_(1000/T) data meet the criterion τ_α_ > 10^−12^ s, previously reported for other representatives of sizable glass-formers and identified as a characteristic feature of their dynamics^3^.

To compare the temperature behavior of the relaxation times for all isomers, an Angell plot was created where the logarithm of relaxation times, τ_α_, τ_*slow*_ and τ_*fast*_, were depicted versus normalized temperature T_g_/T, see panel b Fig. 5. The comparison of their course indicated interesting relationships. It seems that the character of temperature changes reflects the mechanism of reorientations dominating the dielectric response. For the α_*fast*_-process in meta-CF_3_ and α-process in ortho-CF_3,_ very similar behavior was observed. It supports our previous claim that long-axis reorientations determine the reorientation dynamics of ortho-CF_3_ to a large extent. Based on the VFT function, the fragility coefficient *m* was determined. The *m* value refers to the rate at which structural relaxation times deviate from a linear relationship as *T*_g_ is reached^25^. Fragile glass-formers are characterized by a rapid rate of change, while strong substances are characterized by the opposite. To determine the fragility we used the following formula^26,27^:

$$m = \left. {\frac{{dlog\tau_{\alpha } }}{{d\left( {T_{g} /T} \right)}}} \right|_{{T = T_{g} }}$$

The calculated *m* values were relatively similar for all isomers studied. For para isomer *m* = 99. A similar value was found for the α_*slow*_-process in meta-CF_3_, i.e. *m* = 92. For the α-process in ortho isomer and α_*fast*_ in meta-CF_3_ slightly higher values were found, i.e. for, *m* = 100 and *m* = 102, respectively. Referring to a frequently used classification of glass-forming materials as strong (*m* ≥ 30), intermediate (30 < *m* < 100), and fragile (100 ≤ *m*)^25^, the sizable molecules studied herein can be considered as moderately fragile glass-forming molecules. It is worth noting that higher fragility values were observed for modes identified with reorientation around the long axis.

### Glassy dynamics at ambient pressure

Below T_g,_ the dielectric response of isomers was dominated by secondary relaxations. This term covers a diversified class of relaxation processes involving molecular fragments or side-group motion as well as intermolecular processes with connections to structural relaxation and glass transition called Johari-Goldstein (JG) relaxations^28–30^.

**Figure 6:**
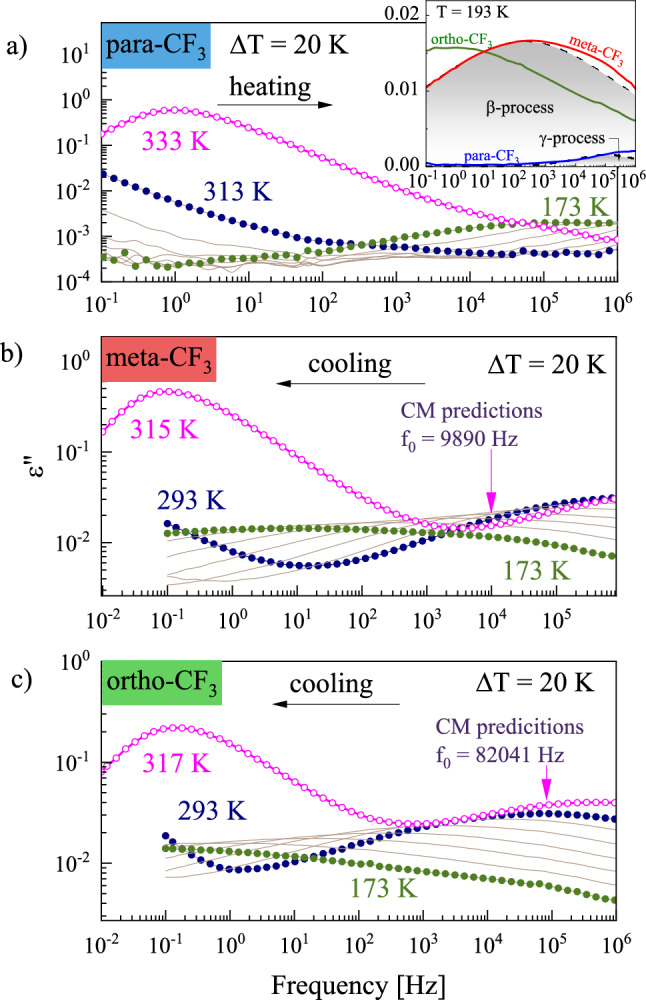
The representative dielectric loss spectra that we recorded for para-CF_3_ (**a**), meta-CF_3_ (**b**), and ortho-CF_3_ (**c**) in the vicinity and below T_g_. For meta and ortho isomers the parameters τ_α_ and β_KWW_ determined from the presented ε"(f) data above T_g_ were used to calculate *f*_0_ (marked with arrow). Inset in panel (**a**) shows a comparison of the loss spectra for glasses at T = 193 K, along with the marked contributions from β and/or γ relaxations (dashed lines) determined from Cole–Cole fits to ε"(f) data for meta-CF_3_.

Playing with the substitution of the polar –CF_3_ group attached to the phenyl ring (Ph-ring) allowed us to indicate with high certainty the molecular origin of the prominent secondary relaxation assigned as β-process. By adding a polar group to the Ph-ring, we have intentionally labeled it and made its internal rotation the dominant contribution to the dielectric response below T_g_. Accordingly, we could relate the strong β-relaxation to the internal rotation of the Ph-ring substituted with the polar –CF_3_ group. The only exception was the para isomer, in which the dipole moment was directed along the long molecular axis (see Fig. 4a) and did not change during the internal rotation of the Ph–CF_3_ unit. Consequently, the corresponding β-process was not visible in the dielectric response of para-CF_3_ (see inset in Fig. 6a).

The dielectric loss spectra collected for an ortho isomer (Fig. 6c) below T_g_ revealed a single secondary β-relaxation related to the internal rotation of the Ph–CF_3_ unit with activation energy of E_a_ = 54.0 ± 0.6 kJ/mol determined from Arrhenius law. For the meta-CF_3_ the rotation of the Ph–CF_3_ unit was faster and associated with a lower activation barrier, i.e. E_a_ = 43.9 ± 0.6 kJ/mol. In comparison to ortho-CF_3_, the internal rotation in the meta-CF_3_ was less coupled to the motion of the rest of the molecule. The proximity of a sizable core hindered the Ph–CF_3_ dynamics in the orto isomer. Interestingly, in the vicinity of T_g_, the time scale of internal rotation in ortho-CF_3_ agrees with the predictions of the Coupling Model (CM). The use of CM is a common way to approximate the Johari-Goldstain (JG) secondary relaxation time identified with intermolecular dynamics and connections to the glass transition^31^. In Fig. 6b, c a vertical arrow indicates the primitive relaxation frequency *f*_0_ = 1/2πτ_0_ corresponding to the primitive relaxation time τ_0_, a precursor of structural relaxation according to CM. We calculated τ_0_ as $\uptau _{0} = \left( {\uptau _{{\text{c}}} } \right)^{{1 -\upbeta _{{{\text{KWW}}}} }} \left( {\uptau _{\upalpha } } \right)^{{\upbeta _{{{\text{KWW}}}} }}$ using τ_c_ = 2 ps, and τ_α_ and β_KWW_ determined for spectra collected above T_g_ and depicted in Fig. 6a, c^31^. For the ortho isomer, the position of the β-process at T = 317 K corresponded well with CM predictions showing that the Ph-CF_3_ motion is coupled with the dynamics of the rest of the molecule. In the meta isomer, the rotating unit was more distant from the sizable core, thus less coupled with core dynamics, and the compliance with the CM predictions was not so good. The agreement between the time scale of primitive relaxation of CM and the β-process provided important information about the sensitivity of Ph-CF_3_ rotational dynamics to the molecular environment. The relevance of intermolecular contributions may result from the presence of fluorine in the rotating fragment, which can interact with hydrogen atoms in its vicinity during rotation. Thus, close to T_g_ the rotation of the Ph-CF_3_ unit will be not independent, but due to intermolecular contacts, will be sensitive to the molecular environment and a liquid-to-glass transition.

In the case of meta-CF_3_, in addition to the β-process, we identified a faster secondary relaxation visible in the loss spectra recorded at low temperatures with E_a_ = 27.4 ± 0.8 kJ/mol, denoted as γ-process (see Fig. 5a). Interestingly, γ-relaxation was also apparent in the loss spectra collected for para-CF_3_ (see inset to Fig. 6a), but its presence was not evidenced in the dielectric response of ortho-CF_3_. The rapid secondary γ-relaxation can be related to alkyl chains, whose dynamics in the case of the ortho isomer might be inhibited by the proximity of the bulky –CF_3_ group. In the para isomer, the γ-process was the fastest, which prevented its detailed analysis in the considered temperature range.

Also noteworthy is the nature of the temperature dependence of relaxation times which vary depending on the molecular origin of a given relaxation mode. In the case of reorientation along different molecular axes, the corresponding relaxation times (α_*slow*_ and α_*fast*_ in meta-CF_3_) separate at high temperatures where the motions are more independent, and merge on cooling. For the β-process opposite behavior was found since it separates systematically from structural relaxation on cooling as the temperature decreases towards T_g_.

### Reorientation dynamics at elevated pressure

**Figure 7:**
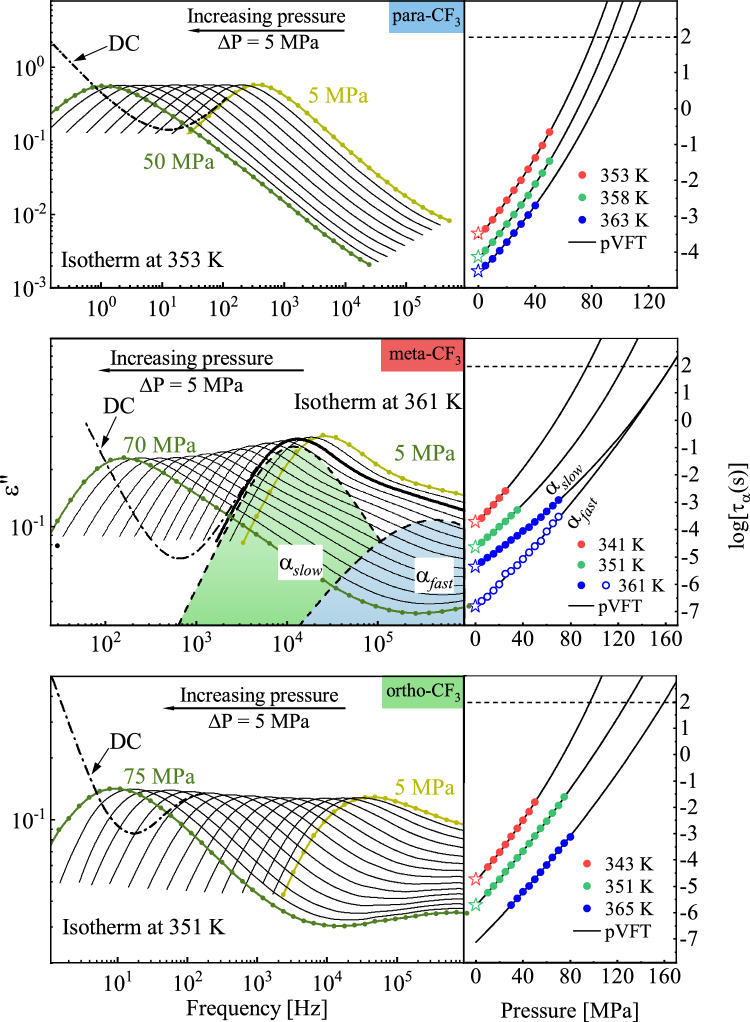
Left panel: representative dielectric loss spectra ε"(f) for selected isotherm measured during compression for indicated pressure ranges with the step of Δp = 5 MPa. The dc- conductivity was subtracted except for one spectrum which illustrates its impact. For meta-CF3 the contribution from α_*slow*_ and α_*fast*_ was indicated. Right panel: pressure dependence of relaxation times τ_α_, τ_*slow*_ and τ_*fast*_ (only for meta-CF_3_). Stars indicate τ_α_ from ambient pressure measurements. The solid line represents pVFT fit functions. The corresponding fitting parameters are shown in Table 2. The glass transition pressure P_g_ was determined for logτ = 2 as marked by a horizontal dashed line.

In the next stage of our study, we investigated how the isomer's reorientation dynamics can be affected by elevated pressure. Isothermal dielectric measurements were carried out for several temperatures, i.e. 353 K, 358 K, 363 K for para-CF_3_, 341 K, 351 K, 361 K for meta-CF_3_, and 343 K, 351 K, 365 K for ortho-CF_3_. The left panel of Fig. 7 shows representative dielectric loss spectra for one isotherm for each isomer.

With increasing pressure, the α-relaxation process shifts toward lower frequencies, as it is observed during isobaric cooling when dynamics slows down when glass transition is approaching. To analyze the data, we used the HN model as before. The pressure dependence of relaxation times for all isotherms is presented in the right panel of Fig. 7. The nonlinear pressure behavior of characteristic relaxation times could be described by the pressure variant of the VFT function (so-called pVFT)^32^:

$$\tau = \tau_{0} exp\frac{{D_{p} P}}{{P_{0} - P}}$$

where τ_0_ is the relaxation time at ambient pressure, P_0_ is the pressure of the ideal glass, and D_p_ is a parameter depending on the pressure fragility of the material. The fitting parameters of the pVFT function are depicted in Table 2.

**Table 2:** Parameters determined from dielectric measurements at elevated pressure characterizing the reorientation dynamics of isomers—the pressure coefficient of glass transition (dT_g_/dP), activation volume at T_g_ (ΔV^#^), fitting parameters of pVFT function (logτ_0_, D_P_, P_0_), the pressure value P_g_ corresponding to the glass transition at various T.

	dT_g_/dP (K/MPa)	ΔV^#^ (cm^3^/mol)	T (K)	logτ_0_ (s)	D_p_	P_0_ (MPa)	P_g_ (MPa)
para-CF_3_	0.387 ± 0.070	735	353	− 3.58 ± 0.01	12.29 ± 1.02	260.96 ± 14.94	81.5
			358	− 4.17 ± 0.01	12.03 ± 0.81	272.89 ± 14.94	92.5
			363	− 4.56 ± 0.02	12.51 ± 3.45	302.95 ± 71.85	104.6
meta-CF_3_	0.332 ± 0.010	648	342	− 3.8 ± 0.01	16.17 ± 6.79	357.46 ± 138.6	94.3
			351	− 4.64 ± 0.01	12.87 ± 3.29	366.7 ± 84.23	124.8
			361	− 5.32 ± 0.01	12.31 ± 1.53	431.19 ± 44.87	160.8
				− 6.82 ± 0.03^α^	80.16 ± 8.63^a^	813.64 ± 69.89^a^	
ortho-CF_3_	0.326 ± 0.011	624	341	− 4.79 ± 0.01	18.87 ± 1.53	366.16 ± 25.22	97.0
			353	− 5.73 ± 0.01	33.21 ± 2.54	680.47 ± 45.98	128.5
			361	− 7.13 ± 0.06	34.15 ± 11.61	757.74 ± 223.67	159.8

^a^Determined for α_*fast*_ process.

**Figure 8:**
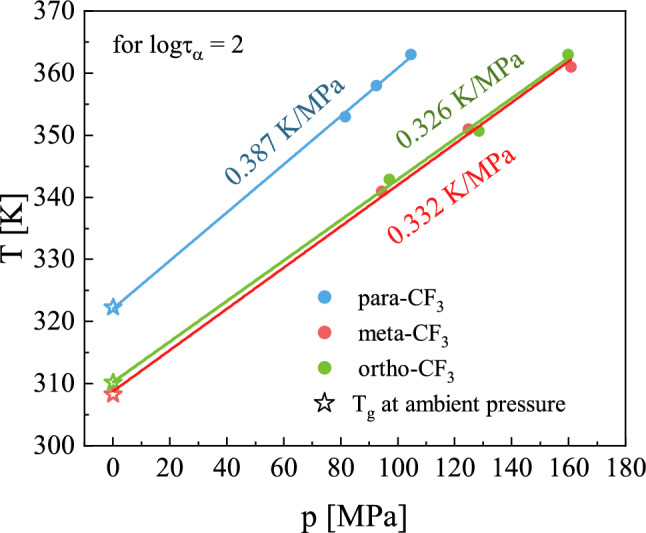
Pressure dependence of the glass transition temperature. Stars denote the T_g_ values obtained from isobaric BDS measurements at ambient pressure, while closed circles represent data from isothermal measurements at elevated pressure. The solid line indicates the linear fit function whose slope was used to determine the dT_g_/dP value. The values of dT_g_/dP for each isomer are depicted.

By extrapolating the pVFT function to logτ = 2, the pressure value corresponding to the glass transition at a given temperature was determined (see Table 2). The sensitivity of glass transition temperature to compression is a characteristic feature of a given material. To characterize it, the pressure coefficient of T_g_ was introduced^5^. For isomers studied herein the applied pressure significantly influenced the glass transition temperature. The T_g_(P) relationship for all isomers, visualized in Fig. 8 had a linear character. In such a case the dT_g_/dP parameter i.e. pressure coefficient of T_g_ can be estimated from the slope of a linear function^33^. The ortho and meta isomers were characterized by almost identical values of dT_g_/dP, i.e. dT_g_/dP = 0.326 and dT_g_/dP = 0.332 K/MPa, respectively, while the para isomer stands out among others with a higher value dT_g_/dP = 0.387 K/MPa. It is worth noting that this is one of the highest values observed to date among non-polymeric glass-forming substances^5,34^. It means that sizable glass formers exhibit an especially pronounced sensitivity of T_g_ to compression.

**Figure 9:**
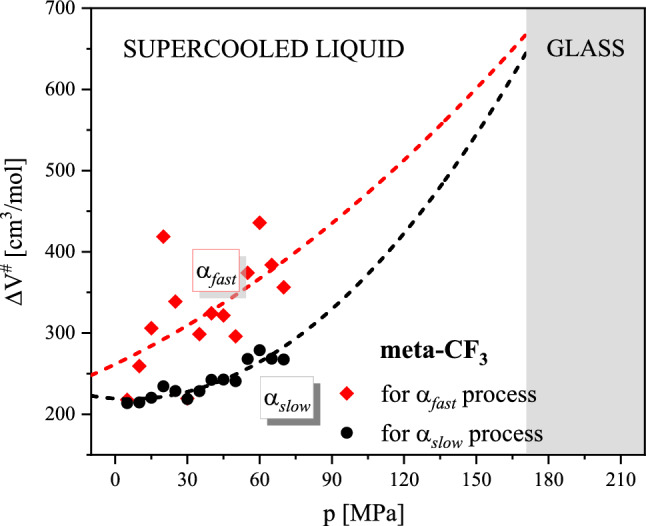
Pressure dependence of activation volume ΔV^#^ for meta-CF_3_ for isotherm collected at T = 361 K in the supercooled liquid state. Dashed lines represent the prediction of activation volumes in higher pressure.

Another important parameter that can be discerned from dielectric data collected as a function of pressure characterizing the pressure sensitivity of relaxation times is activation volume, ΔV^#^. In general terms, the activation volume ΔV^#^ is related to the local volume required for the molecular rearrangement of a relaxing unit. Within the framework of transition state theory, ΔV^#^ is defined as the difference between the volumes occupied by a molecule in activated and inactive states^5^. It is therefore natural to assume that the value of ΔV^#^ should reflect the size of the reorienting objects. Such regularity was illustrated in the past for a series of polyalcohols with increasing van der Waals radius (glycerol, threitol, xylitol, and sorbitol)^35^. For meta-CF_3_ the bifurcation of the structural relaxation process, observed in ambient pressure studies at high T, was successfully observed for isotherm collected at T = 361 K. This allows us to examine the effect of pressure on both relaxations, α_*slow*_ and α_*fast*_. We determined ΔV^#^ value for α_*slow*_ and α_*fast*_ using the following formula:

$$\Delta V^{\# } = RT\left( {\frac{dln\tau }{{dP}}} \right)_{T}$$

where R is the gas constant and T is the temperature^5^. The pressure dependence of ΔV^#^ for both modes in meta-CF_3_ is presented in Fig. 9.

It may be surprising to see that a larger activation volume was found for the α_fast_-process identified previously with the long-axis reorientations. When considering a single rod-like molecule, such rearrangements, intuitively, are regarded as less massive and less volumetric in comparison to short-axis reorientations. Therefore, we expected that in thermodynamic conditions, where particular relaxation modes can be detected, the motions relative to the short-axis would be characterized by a larger activation volume. The opposite behavior that we observed in the experiment can be understood if the molecules form an anisotropic cluster in which the short and long axes can be also distinguished. The larger activation volume for molecular rearrangements along the long axis can be explained when the molecules in the cluster are arranged parallel to each other and create an ellipsoid shape in which the long axis of the molecule becomes the short axis of the cluster. Then, the behavior illustrated in Fig. 9 can be rationalized. Our observation suggests that in the case of anisotropic objects, the activation volume may be a source of information about the morphology of relaxing clusters of dynamically correlated molecules. As the glass transition approaches during compression, a cooperatively relaxing cluster becomes larger and larger and its shape will change. So theoretically the change in activation volume could be an indicator of the cluster morphology at least under certain thermodynamic conditions when the cooperativity onset is not too high. As the pressure increases, the number of cooperatively rearranging molecules will grow limiting our ability to separate different aspects of molecular motion and corresponding ΔV^#^ values.

To compare the activation volume near the glass transition for all isomers we used the formula proposed by Paluch et al.^5,36^:

$$\Delta V^{\# } = 2.303R\left( {\frac{{dT_{g} }}{dP}} \right)m$$

where R is the gas constant, dT_g_/dP is the pressure coefficient of glass transition, and *m* is the fragility parameter determined from isobaric measurements (depicted in Table 1). Knowing that the activation volume calculated for reorientation relative to different molecular axes may yield different values, the choice of a formula allowing to determine ΔV^#^ directly in T_g_ seemed to be the most appropriate. Based on the assumption that individual modes merge in T_g_, such an approach allowed us a reliable comparison of the properties of individual isomers. The calculated activation volumes ΔV^#^ at T_g_ for ortho-CF_3_ and meta-CF_3_ were similar, i.e. ΔV^#^ = 624 cm^3^/mol and ΔV^#^ = 648 cm^3^/mol, respectively. For para-CF_3_ the highest value equal to ΔV^#^ = 735 cm^3^/mol was found. The much higher ΔV^#^ for para-CF_3_ in comparison to other isomers can be due to the most elongated shape of para isomer as illustrated in Fig. 1. Interestingly, the elongation of the molecule caused by the substitution of the CF_3_ group in the para position impacts the value of the ΔV^#^ near T_g_ to such a great extent. Taking into account the highly cooperative nature of molecular motion in the vicinity of T_g_, the eventual lack of differences could be easily rationalized. Meanwhile, the observed distinct behavior of the para-CF_3_ proves that the ΔV^#^ near T_g_ is still highly sensitive to molecular shape and degree of molecule elongation.

The above discussion regarding differences in dynamical behavior near T_g_ in the context of the activation volume and previous discussion about the limited possibility of distinguishing α_*slow*_ and α_*fast*_ modes in meta-CF_3_ near T_g_ prompted us to further studies aimed at better understanding the cooperative nature of dynamics of sizable molecules. The concept of cooperatively rearranging regions (CRRs), was introduced by Adam and Gibbs to describe the relaxation behavior of molecules that can rearrange their configurations independently of the environment^4^. Such structural subunits increase on cooling (or during compression), so as the dynamics evolve towards the glass transition the number of molecules relaxing cooperatively increases. For the systems studied, the increasing degree of motion cooperativity observed on cooling is of great importance because the bimodal character of structural relaxation peak could be distinguished only at high temperatures and low pressures when the motion of molecules was more independent.

**Figure 10:**
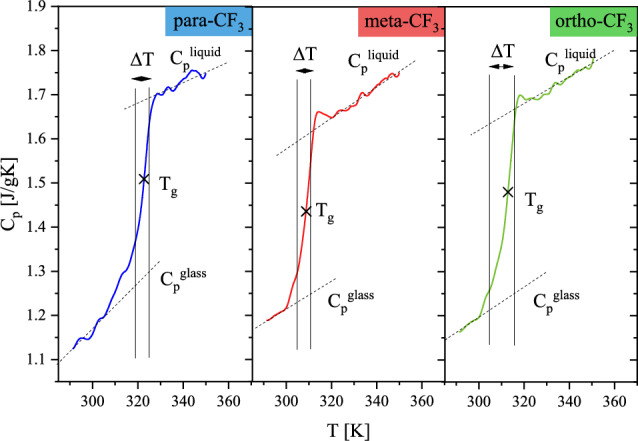
Temperature dependences of quasi-static specific heat capacity C_p_ for the investigated isomers used to calculate the *N*_*α*_^*D*^ (T_g_) using the Donth method.

To calculate a number of dynamically correlated molecules near the glass transition we performed TMDSC measurements and used the approach proposed by Donth^37^. This method is based on the fluctuation–dissipation theorem, which, by studying enthalpy fluctuations, allows us to determine quantitatively the size of the cooperatively rearranging regions. The number of dynamically correlated molecules *N*_*α*_^*D*^ at T_g_ was calculated using the following equation:

$$N_{\alpha }^{D} \left( {T_{g} } \right) = \frac{{N_{A} \left( {\frac{1}{{C_{p}^{glass} }} - \frac{1}{{C_{p}^{liquid} }}} \right)}}{{M\left( {\delta T} \right)^{2} }}k_{B} T_{g}^{2}$$

where N_A_ is Avogadro's number, C_p_^glass^ and C_p_^liquid^ are the heat capacity of glass and liquid at T_g_, k_B_ is Boltzmann's constant, M is the molar mass, and δT is the average temperature fluctuation associated with the glass transition dynamics^38^. The results of TMDSC measurements are shown in Fig. 10. The ΔT range was determined by 16% and 84% of the value of the total difference in heat capacity between glass and liquid. To calculate δT, the value of ΔT was divided by 2.5 as our measurements were performed during heating. The calculated *N*_*α*_^*D*^ values at T_g_ are summarized in Table 3. The highest number of dynamically correlated molecules was found for para-CF_3_, i.e. *N*_*α*_^*D*^ = 55. For meta-CF_3_ and ortho-CF_3_ *N*_*α*_^*D*^ = 44 and *N*_*α*_^*D*^ = 35, respectively. The values obtained for studied isomers are much lower than those reported in the past for different classes of glass-forming liquids. The summary of available data can be found in ref.^39^ showing that reported values cover the range of 70 − 200 for van der Waals liquids and H-bonded liquids and 200 − 820 for polymers. The small values found for sizable isomers can be explained by their large size. Next to size, the molecular stiffness of sizable cores formed by multiple ring-based groups is probably important. It is interesting to note that the variations in *N*_*α*_^*D*^ values apparently depend on the molecular shapes of investigated isomers with the highest *N*_*α*_^*D*^ value for the most elongated isomer.

**Table 3:** Parameters used to calculate the number of dynamically correlated molecules *N*_*α*_^*D*^ at T_g_, according to the Donth approach and the values of *N*_*α*_^*D*^, the volume of correlated molecules V_α_, and radius of correlation ξ determined at T_g_ for investigated isomers.

	C_p_^glass^ (J/g∙K)	C_p_^liquid^ (J/g∙K)	T_g_ (K)	δT (K)	N_α_^D^	V_α_ (nm^3^)	ξ (nm)
para-CF_3_	1.34	1.68	323	1.97	55	48.89	3.81
meta-CF_3_	1.24	1.60	309	2.30	44	39.21	3.54
ortho-CF_3_	1.26	1.65	313	2.68	35	30,68	3.26

**Figure 11:**
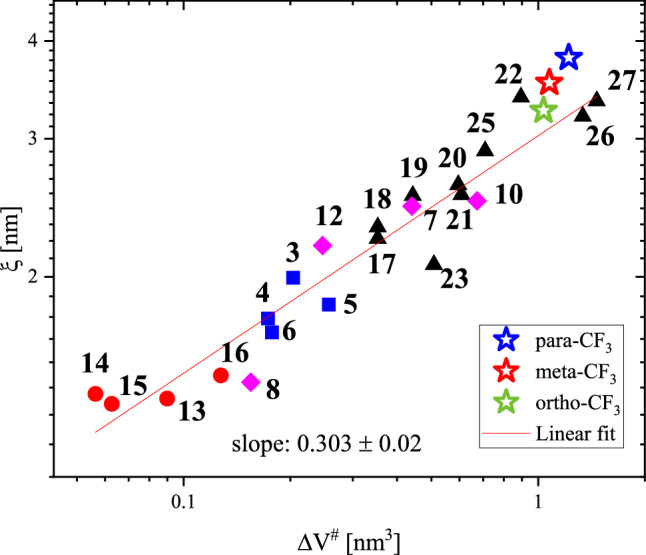
Correlation between the cooperativity length scale ξ and the activation volume ΔV^#^ at T_g_ reported by Hong et al.^40^ with added data for sizable isomers investigated herein (marked with stars). The compounds hidden under the depicted numbers are described in ref.^40^.

Knowing the number of dynamically correlated molecules, we could determine the occupied volume as $V_{\alpha } = N_{\alpha }^{D} M/\rho$ where ρ is a density assumed to be 1.15 g/cm^3^ for all isomers (i.e. value determined from helium pycnometer for meta-CF_3_), and subsequently calculate the radius of correlation using the following relationship $\xi = V_{\alpha }^{1/3}$^40^. The value of *ξ* corresponds to the (typical) size of a CRR postulated by Adam and Gibbs, and mentioned before^4^. Hong et al. showed that there is a correlation between the radius of cooperatively reorientating domains and the activation volume^40^. This correlation was satisfied for a wide and diverse range of materials from low-molecular-weight glass formers to larger but flexible polymers. Thus, it was interesting to check if this correlation is also valid for sizable isomers studied herein. Figure 11 shows the correlation between ξ and ΔV^#^ reported by Hong et al.^40^ with added data for sizable isomers. One can see that our results (marked with stars) matched the trend established by the authors very well. After taking into account data points for sizable molecules, the slope of a linear function that approximates the correlation is equal to 0.303 ± 0.02. The highest activation volume ΔV^#^ near T_g_ was found for para isomer characterized by the highest radius of correlation and the highest volume occupied by dynamically correlated molecules. The characteristic length scale related to dynamic cooperativity reported for various glass-forming liquids was estimated to be 1 – 4 nm^37^. Referring to this, the values obtained for the tested isomers are relatively high. For ortho-CF_3_ the radius of correlation was equal to ξ = 3.26 nm. The higher value was found for meta-CF_3_ i.e. ξ = 3.54 nm while for para-CF_3_ the determined ξ value was the highest, i.e. ξ = 3.81 nm. The increasing correlation length scale determined at T_g_ for particular isomers corresponds well with the increasing degree of molecule elongation resulting from the different positions of the –CF_3_ group, which again proves the overriding role of anisotropy in determining the dynamic properties of isomers studied. Our results i.e. confirmation of correlation compliance for sizable glass-formers pointed out that the ξ parameter has a more universal meaning than the number of correlated molecules. The value of *N*_*α*_^*D*^, determined by the specific and inherent properties of the system, can be treated more as a characteristic feature of a given class of glass-forming molecules.

## Conclusions

In the present work, a series of structural isomers were studied by differential scanning calorimetry and dielectric spectroscopy at ambient and elevated pressure. Investigated isomers possess the same large-sized rigid core and a trifluoromethyl group variously substituted in the ortho, meta, or para positions to the phenylene ring. Variations in the position of the polar –CF_3_ group differentiated the dipole moment of the molecule and the molecular shape. Our results showed that the specific molecular features of isomers studied—their large size, stiffness of some heterocyclic building blocks, elongated shape, the dipole moment properties, affect the picture of molecular dynamics to a great extent. From both calorimetric and dielectric studies, it was shown that isomers differ in their glass transition temperature, melting point, and tendency to recrystallize from the supercooled liquid state. The observation of bifurcation of the structural relaxation process, or the significant frequency distribution of relaxation times, is closely related to the detection of different types of relaxation motions, attributed to long and short axes reorientations of anisotropic molecules studied herein. We confirmed that depending on the thermodynamic conditions and cooperativity degree, the individual modes could be distinguished in the dielectric response of some sizeable glass formers. The meta-CF_3_ isomer is another example of a sizable glass-forming molecule for which it was possible to distinguish the time scale of individual modes associated with long or short axes reorientations at (T, p) conditions where the dynamics were more independent. This is an important observation showing that spectra recorded at high temperatures may contain additional information about motion components that are unattainable around T_g_ due to the highly cooperative nature of molecular dynamics. As in our previous study on another group of sizable glass-formers, unusually large pre-exponential factors τ_0_ were observed^3,7^ which strengthens the general importance of our previous report. Molecular dynamics studies under elevated pressure revealed that the entire series was characterized by very high values of dT_g_/dP and ΔV^#^. The highest values of the mentioned parameters were found for the most elongated para-CF_3_ isomer. An interesting result was the observation that the long-axis reorientations were characterized by a larger activation volume than those relative to the short axis, which was contrary to common-sense expectations. An explanation for this situation may be to consider a cluster of cooperatively reorienting molecules, rather than the result expected from a single molecule. Our discussion shows that the activation volume can contain information about the morphology of relaxing units and how clusters of cooperatively reorientating molecules grow. We identified another feature specific to the class of sizable glass formers, which was small magnitudes of the number of dynamically correlated molecules in T_g_. The determined values of this parameter are the lowest ever reported for van der Waals glass-forming molecules. Interestingly, among so many peculiar properties characterizing the dynamics of sizable isomers, it was found that they satisfied a correlation valid for many other glass-forming systems between the radius of correlation at T_g_ and the activation volume.

Our results clearly show that not only the chemical composition or mass of the molecules but also molecular shape, anisotropy, and the place of substitution of the polar group can affect the reorientation dynamics. This discovery may contribute to a better understanding of the link between the structure of molecules and their dynamics, which is desirable, especially in the context of designing new molecules for selected applications. Further investigations improving our molecular-level understanding of remarkable sizable systems dynamics, e.g. employing Raman or infrared spectroscopy measurements, can bring us closer to the ultimate goal that has remained unchanged for years which is improving the understanding of glass-formers dynamics in general.

## Methods

### Materials

We investigated herein three isomers of sizable molecules whose chemical structures are shown in Fig. 1. All materials were synthesized by TriMen Chemicals (Łódź, Poland) with a declared purity of ≥ 97%. Isomers studied herein will be reffered to as ortho-CF_3_ (*N*-(7-[(2-trifluoromethylphenyl)ethynyl]-9,9-dibutyl-9H-fluoren-2-yl)-*N*,*N*-diphenyamine), meta-CF_3_ (*N*-(7-[(3-trifluoromethylphenyl)ethynyl]-9,9-dibutyl-9H-fluoren-2-yl)-*N*,*N*-diphenyamine), para-CF_3_ (*N*-(7-[(4-trifluoromethylphenyl)ethynyl]-9,9-dibutyl-9H-fluoren-2-yl)-*N*,*N*-diphenyamine. The molecular mass of each compound was M = 613.75 g/mol. Note that the supplier of the para-CF_3_ sample was different than in ref.^7^ and found properties differed slightly from a batch of the material tested elsewhere.

### Differential scanning calorimetry (DSC)

We used a Mettler-Toledo DSC1 STARe differential scanning calorimeter to study the thermal properties and phase transformations in the investigated isomers. This calorimeter was equipped with a nitrogen cooling system and an HSS8 ceramic sensor equipped with 120 thermocouples. Temperature and enthalpy calibration was carried out using indium and zinc standards. The samples (with a mass of around 5 mg) were placed in 40 µl aluminum vessels. The measurement protocol for each sample was the same. In the first temperature ramp, the crystalline sample was heated from T = 273 K to T = 453 K with a heating rate of 10 K/min. After the first scan, the sample was cooled at 10 K/min to vitrify the liquid and measured again on heating. The melting and crystallization temperatures were determined from the onset of the process, while the glass transition temperature was assessed from the midpoint of the heat capacity change.

Stochastic temperature-modulated differential scanning calorimetry (TMDSC) implemented by Mettler-Toledo (TOPEM) was used to determine the calorimetric relaxation times near the glass transition and heat capacity of the studied compounds. The samples were heated at a rate of 0.5 K/min. In the experiment, the temperature amplitude of the pulses of 0.5 K was selected. We analyzed the dynamic behavior in the range from 5 to 20 mHz. The values of the quasi-static heat capacity were calibrated using a sapphire reference curve.

### Broadband dielectric spectroscopy (BDS)

The study of the complex electric permittivity $\varepsilon^{*} \left( f \right) = \varepsilon^{\prime } \left( f \right) - i\varepsilon^{\prime \prime } \left( f \right)$ at varying temperatures was carried out using an Alpha Impedance analyzer spectrometer by Novocontrol. The samples were analyzed in the frequency range of 10^−1^—10^6^ Hz. In the vicinity of the glass transition temperature, T_g_, the frequency range was extended to 10^−2^ Hz. Before the measurement, the materials were dried under vacuum after melting (40 min at T = 363 K) and then quenched on a cooled copper plate to vitrify. The sample has been placed between parallel steel capacitor plates with a diameter of 15 mm. A gap of 0.1 mm was provided by quartz fibers. The capacitor with the investigated material was placed in the spectrometer and analyzed immediately after preparation. Dielectric measurements at ambient pressure were carried out with a step of ΔT = 2 K at various temperature ranges, i.e. 327 K—395 K for para-CF_3_, 313 K—387 K for meta-CF_3_, and 315 K—355 K for ortho-CF_3_. The ortho and meta isomers were measured on cooling while para, due to its high tendency to crystallization, could be measured on heating only. The Novocool system by Novocontrol was responsible for stabilizing the temperature to within 0.2 K.

Dielectric measurements under elevated pressure were carried out by combining an Alpha-A impedance analyzer (range 10^−1^ to 10^6^) by Novocotrol with a hydrostatic pressure-generating system by Unipress, a tensometer by Nova Swiss, and a thermostat by Julabo. A set temperature (± 1 K) was reached by flowing liquid in a heating jacket around the pressure chamber. The investigated samples were prepared in the same manner as in the dielectric measurements at ambient pressure by melting, drying under vacuum, and then quenching on a cooled copper plate. Materials were placed between two parallel steel capacitor plates (with a diameter of 15 mm) separated with a 0.1 mm thick Teflon ring. Two wires connected the capacitor covers with the analyzer. After preparation, the sample was secured with Teflon tape and placed inside a chamber filled out with non-polar silicone oil as the compression medium.

## Data Availability

Data supporting the findings of this study are available within the article. Source data are available from the corresponding author on request.
